# Racial disparities in post-operative complications and discharge destination following total joints arthroplasty: a national database study

**DOI:** 10.1007/s00402-022-04485-3

**Published:** 2022-06-13

**Authors:** Alex Upfill-Brown, Noah Paisner, Adam Sassoon

**Affiliations:** 1 Department of Orthopaedic Surgery, David Geffen School of Medicine at UCLA, 1225 15thSt, Suite 3145, Santa Monica, CA 90404 USA; 2 Pacific Northwest University School of Health Sciences, Yakima, WA USA

**Keywords:** Total knee arthroplasty, Total hip arthroplasty, Racial disparities, Post-operative complications, Discharge destination

## Abstract

**Introduction:**

The objective of this study was to explore race-based differences in 30-day complication rates following total joint arthroplasty (TJA) using a large national database.

**Methods:**

Patients undergoing primary, elective THA and TKA between 2012 and 2018 were retrospectively reviewed using the ACS-NSQIP. We compared Black and Hispanic patients with non-Hispanic White patients using multivariate statistical models adjusting for demographic, operative, and medical characteristics.

**Results:**

A total of 324,795 and 200,023 patients undergoing THA and TKA, respectively, were identified. After THA, compared to White patients, Black and Hispanic patients were more likely to be diagnosed with VTE (*p* < 0.001), receive a blood transfusion (*p* < 0.001), and to be discharged to an inpatient facility (*p* < 0.001). After TKA, compared to White patients, Black and Hispanic patients were more likely to experience a major complication (*p* < 0.001 and *p* = 0.008, respectively), be diagnosed with VTE (*p* < 0.001), and be discharged to a facility (*p* < 0.001).

**Conclusions:**

Our findings indicate higher rates of VTE, blood transfusions, and discharge to an inpatient facility for Black and Hispanic patients when compared to White patients following TJA, though we are unable to comment on the etiology of these disparities. These results may contribute to a growing divide with respect to outcomes and access to TJA for these at-risk patient populations.

**Supplementary Information:**

The online version contains supplementary material available at 10.1007/s00402-022-04485-3.

## Introduction

Research on racial inequities in total joint arthroplasty (TJA) so far has been consistent with broader trends well-described in other domains of social determinants of health research. The experience of a Black or Hispanic patient undergoing TJA is significantly different than that of a White patient, in terms of both reduced utilization and poorer outcomes [1–4].

It is well documented in the literature that Black patients have lower rates of utilization of TJA compared to White patients [4–6]. Skinner et al. [6] found the annual rate of knee arthroplasty was higher for non-Hispanic White men and women (4.82 and 5.97 procedures per 1000, respectively) than for Hispanic men and women (3.46 and 5.37 per 1000, respectively) and Black men and women (1.82 and 4.84 per 1000, respectively). Despite the reduced utilization, Black individuals have been shown to have higher rates of symptomatic knee and hip osteoarthritis [7]. For example, Jordan et al. [8] found that severe radiographic knee osteoarthritis was twice as frequent in Black patients compared to White patients. In terms of outcomes, Black patients have been shown to have worse 2-year pain and function scores [1], are more likely to be discharged to a skilled nursing facility [9, 10], have higher rates of readmission [11], higher rates of infection [2], and higher rates of revision [12, 13] compared to White patients. Similar data exist regarding Hispanic patients, who have also been shown to have reduced rates of utilization of TJA [3], higher rates of post-operative complications [14], and are more likely to have a non-home discharge [15] when compared to White patients.

While prior studies have highlighted the existence of racial disparities in TJA outcomes, few have discussed actions to improve these. The purpose of our study was to thoroughly explore the effect of race on 30-day complication rates following total hip arthroplasty (THA) and total knee arthroplasty (TKA) using a large database. Our hypothesis was that complication rates would be higher for Black and Hispanic patients when compared to White patients. Specifically, we hypothesized that rates of venous thromboembolism, blood transfusion, surgical site complications, and discharge to a facility will be higher in non-White patients compared to White patients.

## Materials and methods

We utilized the American College of Surgeons National Surgical Quality Improvement Program (NSQIP) database to identify patients undergoing a primary hip or knee arthroplasty between January 1, 2012 and December 31, 2018. Current Procedural Terminology (CPT) codes were used to identify patients undergoing primary total hip arthroplasty (THA) (27,130) or primary total knee arthroplasty (TKA) 27,447). The NSQIP includes data from over 700 hospitals and is assembled by hospital-appointed, specially trained staff members. The NSQIP database includes data regarding baseline patient demographics, surgical details, and 30-day post-operative outcomes. The data collection process is overseen by a surgeon champion, and independent reviews found overall data reliability to be excellent.

Patient characteristics collected from the registry included patient age, sex, height, weight, smoking history (within 1 year), American Society of Anesthesiologists (ASA) class, operative time (in minutes), and medical comorbidities including diabetes, chronic obstructive pulmonary disease (COPD), liver disease with ascites, congestive heart failure (CHF), hypertension (HTN), sepsis within 48 h of surgery, bleeding disorders, chronic steroid use for medical condition, disseminated cancer, and dialysis-dependent kidney disease. Body mass index (BMI) was calculated from each patient’s height and weight. Functional status was defined as the patient’s ability to perform the activities of daily living (ADLs) in three categories. These categories included ADLs performed independently, in a partially dependent manner, or a completely dependent manner within the 30 days prior to admission.

Data on postoperative medical complications within 30 days were collected. Primary outcomes of the study were major complications, mortality, return to the operating room (OR), venous thromboembolism (VTE, including pulmonary embolism (PE) or deep vein thrombosis (DVT)), surgical site complications (including deep infection, wound infection, superficial infection or dehiscence), post-operative blood transfusion and discharge destination (facility versus home). Major complications were defined as the occurrence of any of the following: death, on ventilator more than 48 h, unplanned intubation, stroke/cerebrovascular accident, DVT, PE, cardiac arrest, myocardial infarction (MI), acute renal failure (ARF) requiring dialysis, sepsis, septic shock, return to OR, wound dehiscence, superficial infection, wound infection, deep surgical organ/space infection.

Baseline characteristics of patients undergoing primary total joint arthroplasty were summarized using descriptive statistics. Patients undergoing TKA and THA were analyzed separately. Multivariate logistic regression was used to estimate the relationship between patient reported race and outcomes of interest, adjusted for age, gender, BMI, ASA class, OR time, and medical comorbidities. Standardized odds ratio (OR), 95% confidence intervals (CI), and p-values were computed. Model-adjusted complication rates and confidence intervals generated using 1000 bootstrapped samples for different reported racial groups at means of independent variables. Patients with missing covariates were excluded from multivariate analysis. Statistical significance was defined as p < 0.05. Statistical analyses were performed using R 3.6.0 (R Foundation for Statistical Computing, Vienna, Austria).

## Results

### Demographic characteristics

A total of 200,023 and 324,795 patients undergoing TKA and THA, respectively, were identified between 2012 and 2018 (Table 1). TKA patients were more likely to be older, have a higher BMI and be from higher ASA classes. THA patients had a higher probability of smoking than TKA patients, while TKA patients had higher rates of diabetes and hypertension than THA patients.

**Table 1:** Baseline demographics of patients undergoing primary THA and TKA in study period

	THA	TKA	*P* value
	*n* = 200,023	*n* = 324,795	
Sex, Female	55.1% (110,174)	61.6 (200,160)	< 0.001
Age			
0–50	8.2% (16,425)	3.5% (11,235)	< 0.001
50–65	37.8% (75,574)	36.3% (117,953)	
65–80	43.2% (86,370)	51.0% (165,785)	
80+	10.8% (21,653)	9.2% (29,822)	
Race			
White	72.8% (145,665)	70.6% (229,239)	< 0.001
Hispanic	2.9% (5863)	5.1% (16,603)	
Black	7.5% (14,974)	7.5% (24,370)	
Other	2.1% (4120)	2.9% (9411)	
Missing	14.7% (29,401)	13.9% (45,172)	
BMI			
Mean (SD)	30.2 (6.3)	33.0 (6.8)	< 0.001
ASA			
1	3.9% (7705)	1.9% (6193)	< 0.001
2	52.7% (105,315)	48.8% (158,470)	
3	41.3% (82,488)	47.6%(154,373)	
4+	2.1% (4259)	1.7% (5399)	
Functional status			
Independent	97.9% (195,220)	98.9% (319,424)	< 0.001
Not independent	2.1% (4119)	1.1% (3698)	
OR time			
Mean (SD)	92.0% (39.4)	91.9% (37.0)	0.35
Discharge destination			
Facility	19.2% (38,343)	20.7% (257,412)	< 0.001
Home	80.8% (161,369)	79.3% (67,110)	
Medical comorbidities			
Smoker	12.8% (25,646)	8.3% (26,899)	< 0.001
Diabetes	12.1% (24,191)	18.1% (58,846)	< 0.001
HTN	55.5% (110,982)	64.8% (210,375)	< 0.001
COPD	4% (7995)	3.5% (11,254)	< 0.001
CHF	0.4% (746)	0.3% (966)	< 0.001
Dialysis	0.3% (527)	0.2% (521)	< 0.001
Steroids	3.8% (7664)	3.6% (11,540)	< 0.001
Systemic infection	0.5% (1023)	0.2% (623)	< 0.001
Bleeding disorder	2.3% (4608)	2.1% (6821)	< 0.001
Metastatic cancer	0.4% (778)	0.1% (355)	< 0.001

Of THA patients, 7.5% identified as Black and 2.9% identified as Hispanic while 2.1% identified as another non-White racial group and 14.7% of patients did not report (Table 1). Comparatively, 7.5% of TKA patients identified as Black and 5.1% identified as Hispanic. A further 2.9% reported another racial identity, while 13.9% of patients did not report.

### Unadjusted complication rates

Overall, 10.8% of THA patients and 7.8% of TKA patient experienced any complication in the first 30 days following surgery (Table 2). Blood transfusion was the most common complication occurring in 6.9% of THA patient and 3.9% of TKA patients. Return to the OR occurred in 2.0% of THA patients and 1.15% of TKA patients. VTE occurred at a higher rate following TKA, in 1.2% of patients compared to 0.6% of THA patients. Surgical site complications occurred at higher rate following THA, in 1.2% of patients compared to 1.0% of TKA patients.

**Table 2:** 30-day unadjusted complication rates for all primary THA and TKA patients

	THA	TKA
	*n* = 200,023	*n* = 324,795
Any complication	10.84% (21,673)	7.8% (25,329)
Major complication	3.29% (6583)	2.91% (9440)
Unplanned re-admit	2.31% (4618)	1.69% (5474)
Return to OR	1.99% (3985)	1.15% (3746)
Reintubation	0.17% (339)	0.14% (448)
On Vent > 48 h	0.07% (145)	0.06% (201)
Death	0.2% (395)	0.1% (327)
Cardiac arrest	0.09% (183)	0.08% (255)
MI	0.26% (514)	0.2% (642)
CVA	0.1% (191)	0.08% (257)
Dialysis	0.06% (111)	0.05% (178)
Sepsis	0.27% (532)	0.19% (614)
Septic shock	0.06% (124)	0.05% (169)
PNA	0.36% (722)	0.32% (1042)
AKI	0.09% (190)	0.11% (348)
UTI	0.93% (1866)	0.76% (2467)
VTE		
PE	0.26% (523)	0.54% (1764)
DVT	0.38% (754)	0.78% (2540)
Surgical site complications		
Dehiscence	0.11% (229)	0.21% (669)
Infection, superficial	0.6% (1196)	0.52% (1705)
Infection, wound	0.25% (496)	0.12% (401)
Infection, deep	0.28% (566)	0.19% (610)
Blood transfusion	6.91% (13,829)	3.91% (12,691)

### Multivariate analysis of THA complications by racial group

Model adjusted complication rates for complications of interest for THA patients are in Table 3. Model results for each associated model are in supplementary tables S1-S7. In multivariate models there were no differences between White patients and Black and Hispanic patients with regard to rates of major complication, death, return to the operating room or surgical site complications.

**Table 3:** THA multivariate model-adjusted post-operative outcome rates by racial group

	White		Black			Hispanic		
	%^a^	CI^b^	%^a^	CI^b^	*p* value^c^	%^a^	CI^b^	*p* value^c^
Major complication	2.83	(2.74–2.92)	2.78	(2.55–3.04)	0.740	2.89	(2.51–3.33)	0.762
Death	0.07	(0.06–0.09)	0.07	(0.04–0.1)	0.692	0.06	(0.03–0.13)	0.697
Return to OR	1.82	(1.75–1.89)	1.60	(1.43–1.8)	0.037	1.60	(1.32–1.93)	0.192
Surgical site complications	0.86	(0.81–0.91)	0.80	(0.69–0.93)	0.332	0.84	(0.66–1.08)	0.862
VTE	0.50	(0.46–0.54)	0.77	(0.64–0.93)	< 0.001	0.83	(0.63–1.09)	< 0.001
Transfusion	5.27	(5.15–5.39)	6.19	(5.84–6.57)	< 0.001	6.87	(6.3–7.5)	< 0.001
Discharge to facility	15.27	(15.06–15.47)	23.79	(23.04–24.55)	< 0.001	22.32	(21.17–23.52)	< 0.001

^a^Model-adjusted estimated complication rate

^b^Predicted confidence intervals

^c^Estimated *p* value from multivariate regression model

Black patients were more likely to be diagnosed with a VTE in the peri-operative period (OR 1.56, *p* < 0.001, Table S4) with an adjusted mean rate of 0.77% (95% CI 0.64–0.91%) compared to 0.49% (CI 0.46–0.53%) of White patients (Table 3). Similarly, Hispanic patients were at increased risk for VTE (OR 1.68, *p* < 0.001) with an adjusted mean rate of 0.82% (CI 0.59–1.06%).

Black and Hispanic patients were also more likely to receive a blood transfusion after THA (OR 1.19 and 1.33, respectively, *p* < 0.001 for both, Table S6) with adjusted mean rates of 6.17% (CI 5.82–6.54%) and 6.87% (CI 6.33–7.48%), respectively, compared to 5.26% (CI 5.15–5.38%) for White patients.

Finally, Black and Hispanic patients were more likely be discharge to a facility than White patients (OR 1.73 and 1.60 respectively, *p* < 0.001 for both, Table S7). Adjusted mean rates of discharge to facility were 15.22% (CI 15.02–15.42%) for White patients, 23.69% (CI 22.88–24.46%) for Black patients and 22.28% (CI 21.13–23.41%) for Hispanic patients.

### Multivariate analysis of TKA complications by racial group

Model adjusted complication rates for complications of interest for TKA patients are in Table 4. Model results for each associated model are in supplementary tables S8-S14. In multivariate models there were no difference between White patients and Black and Hispanic patients with regard to rates of death, return to the operating room or surgical site complications following TKA.

**Table 4:** TKA multivariate model-adjusted post-operative outcome rates by racial group

	White		Black			Hispanic		
	%^a^	CI^b^	%^a^	CI^b^	*p* value^c^	%^a^	CI^b^	*p* value^c^
Major complication	2.65	(2.58–2.71)	3.28	(3.06–3.5)	< 0.001	2.98	(2.74–3.25)	0.008
Death	0.06	(0.05–0.07)	0.08	(0.05–0.12)	0.233	0.08	(0.05–0.12)	0.348
Return to OR	1.05	(1.01–1.1)	1.19	(1.07–1.33)	0.032	1.04	(0.9–1.2)	0.876
Surgical site complications	0.75	(0.72–0.79)	0.73	(0.65–0.83)	0.690	0.78	(0.66–0.92)	0.707
VTE	1.12	(1.08–1.17)	1.62	(1.47–1.8)	< 0.001	1.46	(1.29–1.65)	< 0.001
Transfusion	3.47	(3.4–3.55)	3.91	(3.69–4.15)	< 0.001	3.45	(3.2–3.72)	0.859
Discharge to facility	18.73	(18.56–18.9)	27.28	(26.68–27.88)	< 0.001	23.54	(22.88–24.21)	< 0.001

^a^Model-adjusted estimated complication rate

^b^Predicted confidence intervals

^c^Estimated *p* value from multivariate regression model

Black patients were more likely to experience major complications compared to White patients (OR 1.25, *p* < 0.001, Table S8). Similarly, Hispanic patients had higher rates of major complications than White patients (OR 1.13, *p* = 0.008). Mean adjusted rates of major complications following TKA were 2.65% (CI 2.57–2.71%), 3.28% (CI 3.06–3.49%) and 2.98% (CI 2.73–3.25%) for White, Black and Hispanic patients, respectively (Table 4).

Black patients were more likely than White patients to be diagnosed with a VTE in the peri-operative period (OR 1.45, *p* < 0.001, Table S11) with an adjusted mean rate of 1.63% (CI 1.46–1.79%) compared to 1.12% (CI 1.08–1.16%) of White patients (Table 4). Similarly, Hispanic patients were at increased risk for VTE (OR 1.3, *p* < 0.001) with an adjusted mean rate of 1.46% (CI 1.28–1.64%).

Black patients, but not Hispanic patients, were at increased risk of post-operative blood transfusion following TKA compared to White patients (OR 1.13, *p* < 0.001, Table S13). Adjusted mean rates of post-operative blood transfusion were 3.48% (CI 3.39–3.56%), 3.92% (CI 3.69–4.14%) and 3.44% (CI 3.17–3.71%) for White, Black and Hispanic patients, respectively.

Black and Hispanic patients were significantly more likely to be discharge to a facility than White patients (OR 1.62 and 1.34 respectively, *p* < 0.001 for both, Table S14). Adjusted mean rates of discharge to facility were 18.72% (CI 18.55–18.87%) for White patients, 27.25% (CI 26.65–27.86%) for Black patients and 23.52% (CI 22.88–24.16%) for Hispanic patients.

## Discussion

After examining the effect of race on 30-day post-operative complication rates following TJA, we found that Black and Hispanic patients had significantly higher rates of VTE than White patients following both THA and TKA. Black and Hispanic patients also had higher rates of post-operative blood transfusion than White patients after THA, while only Black patients had higher rates of this than White patients after TKA. Moreover, Black and Hispanic patients were also found to have a higher rate of major complications following TKA. Finally, Black and Hispanic patients were more likely to be discharged to a facility rather than home than White patients following THA and TKA. There were no consistent differences between groups with regards to death, return to OR, or surgical site complications.

This study is not without limitations. Importantly, NSQIP does not collect information on socioeconomic, insurance status, or behavioral factors (such as smoking), all which could be drivers of racial disparities uncovered in this study. Health disparities are inherently complex and likely due to a multitude of societal and systemic factors, making it difficult to identify a root cause for our findings. Additionally, we were only able to collect data regarding complications on the first 30 days following TJA. Despite these limitations, the NSQIP has been shown to be a trusted means of measuring post-operative complications [16].

Our finding of racial disparities in discharge disposition is in line with previous studies that examined this issue [9]. Higher rates of discharge to an inpatient facility may further disincentivize surgeons from offering TJA to patients requiring discharge to a facility. Finklestein et al. [17] found that discharge to post-acute care (PAC) facilities following TJA decreased in the year following institution of Medicare’s bundled payment program. They also suggested that reductions in PAC usage are the first-line responses of health systems to changes in payment mechanisms. In practice, this could lead to patients requiring PAC placement getting selected against in an effort to control costs. This is especially problematic for Black patients who already have historically lower rates of utilization of TJA when compared to White patients [18]. Addressing disparities in discharge disposition should start with a concerted effort to identify patients at risk and find ways to avoid non-home discharge. While the decision to go to a SNF involves patient factors such as housing and access to assistance, interventions such as postoperative mobility programs and preoperative screening with the Risk Assessment and Prediction Tool (RAPT) may reduce SNF utilization after TJA [19]. Discharge to a facility following TJA is associated with poor outcomes such as higher odds of hospital readmission [20] and increased morbidity [21], so any initiative that can reduce this disparity should be considered.

We find significantly increased rates of post-operative blood transfusions for Black patients following THA and TKA relative to White patients. It has been shown that Black patients may be more likely to undergo TKA at a low-quality hospital [22], which could lead to an increased risk for surgical complications, leading to a greater need for blood transfusions. Additionally, there is evidence to suggest that patients who require blood transfusion or treatment dose anticoagulation in the perioperative period may be at higher risk for periprosthetic joint infection [23, 24].

Both Black and Hispanic patients in our study had increased rates of VTE following both TKA and TJA compared to White patients, distinct from prior studies which have documented a higher VTE risk for Black patients [25]. Black and Hispanic patients report less satisfaction in communication with their physician [14] and have been shown to have lower levels of health literacy [26], which could increase the risk for nonadherence to DVT prophylaxis recommendations. Novel communication strategies or changes to anticoagulation protocols are necessary to improve DVT prophylaxis adherence and eliminate the disparity in VTE rates following TJA. Oral anticoagulants such as aspirin or rivaroxaban may increase patient compliance compared to subcutaneous agents, i.e. enoxaparin.

Another key point regarding our findings lies in what they may indicate given a longer follow-up period. As mentioned above, discharge to a facility, blood transfusion requirement, and VTE following TJA are all associated with poor outcomes such as increased morbidity and periprosthetic infection [24, 25]. Thus, while our follow-up is limited to 30 days, these outcome measures may herald further widening disparities between these groups if longer follow-up was pursued. Additionally, these findings do not occur in isolation, and synergy of these complications, combined with health care access issues and post-acute care disparities, may contribute to downstream effects as well.

It is important to consider that race may be acting as a proxy for socioeconomic status (SES) and insurance status. We cannot explore the effects of either of these individually using the NSQIP, but given their historical linkage with race [27–30], there may be a potential effect. There is evidence to suggest that SES and insurance status are independent risk factors for worse outcomes following TJA [31–33], so it is plausible that these are contributing to the observed findings. This association also speaks to the systemic nature of racial inequities in healthcare, which is important to note in any analysis of race-based differences in healthcare outcomes. Structural changes to healthcare will likely be necessary to improve these disparities and should be the target of future research and policy.

## Conclusion

Our findings indicate that compared to White patients, Black and Hispanic patients have higher rates of VTE, blood transfusions, discharge to an inpatient facility, and major complications following TKA, THA, or both. The etiologies of these disparities are complex and likely stem from reduced access to care leading to delayed presentations, poorer quality care, poorer clinician-patient communication and inadequate post-hospital support systems. Further study in necessary to elucidate how access, in-hospital and post-hospital care contribute to disparities in complications following TJA and identify policy targets that can mitigate these shortcomings.

## Supplementary Information

Below is the link to the electronic supplementary material.

Supplementary file1 (DOCX 47 KB)
