# Supplementary material for: Racial disparities in post-operative complications and discharge destination following total joints arthroplasty: a national database study
Source: Arch Orthop Trauma Surg. 2022 Jun 13;143(4):2227–33. doi: 10.1007/s00402-022-04485-3 (PMC10030399; doi:10.1007/s00402-022-04485-3)
Supplement: Supplementary file 1 — Supplementary file1 (DOCX 47 KB) [file 402_2022_4485_MOESM1_ESM.docx]

**Table S1 – THA multivariate regression model results for major complication**

| **Variable** | **OR** | **CI** | **P val** |
| --- | --- | --- | --- |
| Race |  |  |  |
| *Black* | 0.98 | (0.89, 1.08) | 0.740 |
| *Hispanic* | 1.02 | (0.88, 1.19) | 0.762 |
| *Other* | 0.9 | (0.74, 1.09) | 0.280 |
| *Missing* | 1.01 | (0.93, 1.09) | 0.856 |
| Female | 1.03 | (0.98, 1.08) | 0.285 |
| Age (yrs) | 1.02 | (1.02, 1.03) | <0.001 |
| BMI | 1.02 | (1.02, 1.02) | <0.001 |
| ASA | 1.64 | (1.56, 1.72) | <0.001 |
| Operative Time | 1.04 | (1.04, 1.05) | <0.001 |
| Med History |  |  |  |
| *Smoker* | 1.38 | (1.28, 1.48) | <0.001 |
| *HTN* | 1.1 | (1.04, 1.17) | <0.001 |
| *CHF* | 2.37 | (1.91, 2.95) | <0.001 |
| *DM* | 1.09 | (1.02, 1.17) | 0.012 |
| *Dialysis* | 2.31 | (1.75, 3.06) | <0.001 |
| *Malignancy* | 2.62 | (2.07, 3.32) | <0.001 |
| *Systemic Steroids* | 1.37 | (1.23, 1.53) | <0.001 |
| *Bleeding Disorder* | 1.63 | (1.45, 1.83) | <0.001 |
| *Systemic Infection* | 2.64 | (2.15, 3.23) | <0.001 |

**Table S2 – THA multivariate regression model results for mortality**

| **Variable** | **OR** | **CI** | **P val** |
| --- | --- | --- | --- |
| Race |  |  |  |
| *Black* | 0.92 | (0.59, 1.42) | 0.692 |
| *Hispanic* | 0.87 | (0.44, 1.74) | 0.697 |
| *Other* | 0.5 | (0.18, 1.37) | 0.171 |
| *Missing* | 0.76 | (0.54, 1.07) | 0.115 |
| Female | 0.72 | (0.58, 0.89) | 0.002 |
| Age (yrs) | 1.06 | (1.05, 1.08) | <0.001 |
| BMI | 0.95 | (0.93, 0.97) | <0.001 |
| ASA | 4.41 | (3.64, 5.33) | <0.001 |
| Operative Time |  |  |  |
| Med History | 1.03 | (1.01, 1.05) | 0.005 |
| *Smoker* | 1.26 | (0.9, 1.78) | 0.173 |
| *HTN* | 0.93 | (0.73, 1.19) | 0.562 |
| *CHF* | 4.11 | (2.71, 6.25) | <0.001 |
| *DM* | 0.92 | (0.69, 1.23) | 0.579 |
| *Dialysis* | 4.1 | (2.36, 7.11) | <0.001 |
| *Malignancy* | 4.05 | (2.24, 7.32) | <0.001 |
| *Systemic Steroids* | 0.75 | (0.46, 1.21) | 0.229 |
| *Bleeding Disorder* | 1.78 | (1.28, 2.47) | <0.001 |
| *Systemic Infection* | 5.99 | (4.06, 8.84) | <0.001 |

**Table S3 – THA multivariate regression model results for return to OR**

| **Variable** | **OR** | **CI** | **P val** |
| --- | --- | --- | --- |
| Race |  |  |  |
| *Black* | 0.88 | (0.78, 0.99) | 0.037 |
| *Hispanic* | 0.88 | (0.72, 1.07) | 0.192 |
| *Other* | 1 | (0.79, 1.26) | 0.991 |
| *Missing* | 0.91 | (0.82, 1) | 0.052 |
| Female | 1.14 | (1.06, 1.21) | <0.001 |
| Age (yrs) | 1.01 | (1.01, 1.01) | <0.001 |
| BMI | 1.03 | (1.03, 1.04) | <0.001 |
| ASA | 1.43 | (1.35, 1.52) | <0.001 |
| Operative Time | 1.04 | (1.04, 1.05) | <0.001 |
| Med History |  |  |  |
| *Smoker* | 1.54 | (1.4, 1.68) | <0.001 |
| *HTN* | 1.17 | (1.09, 1.26) | <0.001 |
| *CHF* | 1.85 | (1.35, 2.53) | <0.001 |
| *DM* | 1 | (0.91, 1.09) | 0.977 |
| *Dialysis* | 1.58 | (1.03, 2.41) | 0.034 |
| *Malignancy* | 2.08 | (1.5, 2.88) | <0.001 |
| *Systemic Steroids* | 1.52 | (1.33, 1.74) | <0.001 |
| *Bleeding Disorder* | 1.42 | (1.21, 1.67) | <0.001 |
| *Systemic Infection* | 1.6 | (1.17, 2.21) | 0.003 |

**Table S4 – THA multivariate regression model results for VTE**

| **Variable** | **OR** | **CI** | **P val** |
| --- | --- | --- | --- |
| Race |  |  |  |
| *Black* | 1.56 | (1.28, 1.9) | <0.001 |
| *Hispanic* | 1.68 | (1.26, 2.24) | <0.001 |
| *Other* | 0.65 | (0.38, 1.11) | 0.113 |
| *Missing* | 1.1 | (0.93, 1.31) | 0.263 |
| Female | 1.04 | (0.92, 1.17) | 0.532 |
| Age (yrs) | 1.03 | (1.02, 1.03) | <0.001 |
| BMI | 1.01 | (1, 1.02) | 0.006 |
| ASA | 1.39 | (1.25, 1.56) | <0.001 |
| Operative Time | 1.03 | (1.02, 1.04) | <0.001 |
| Med History |  |  |  |
| *Smoker* | 0.84 | (0.68, 1.03) | 0.085 |
| *HTN* | 0.86 | (0.76, 0.99) | 0.027 |
| *CHF* | 0.49 | (0.18, 1.35) | 0.164 |
| *DM* | 1.06 | (0.9, 1.26) | 0.479 |
| *Dialysis* | 1.22 | (0.53, 2.78) | 0.637 |
| *Malignancy* | 3.78 | (2.42, 5.9) | <0.001 |
| *Systemic Steroids* | 1.18 | (0.9, 1.54) | 0.220 |
| *Bleeding Disorder* | 1.62 | (1.23, 2.15) | <0.001 |
| *Systemic Infection* | 2.02 | (1.21, 3.37) | 0.007 |

**Table S5 – THA multivariate regression model results for surgical site complications**

| **Variable** | **OR** | **CI** | **P val** |
| --- | --- | --- | --- |
| Race |  |  |  |
| *Black* | 0.93 | (0.79, 1.08) | 0.332 |
| *Hispanic* | 0.98 | (0.76, 1.26) | 0.862 |
| *Other* | 0.91 | (0.65, 1.28) | 0.594 |
| *Missing* | 1.87 | (1.68, 2.08) | <0.001 |
| Female | 1.12 | (1.03, 1.22) | 0.009 |
| Age (yrs) | 1 | (1, 1.01) | 0.711 |
| BMI | 1.06 | (1.06, 1.07) | <0.001 |
| ASA | 1.5 | (1.38, 1.62) | <0.001 |
| Operative Time | 1.05 | (1.04, 1.05) | <0.001 |
| Med History |  |  |  |
| *Smoker* | 1.6 | (1.43, 1.79) | <0.001 |
| *HTN* | 1.13 | (1.03, 1.25) | 0.011 |
| *CHF* | 1.94 | (1.29, 2.92) | 0.001 |
| *DM* | 1.11 | (0.99, 1.24) | 0.062 |
| *Dialysis* | 0.57 | (0.24, 1.33) | 0.185 |
| *Malignancy* | 1.18 | (0.68, 2.04) | 0.558 |
| *Systemic Steroids* | 1.62 | (1.37, 1.92) | <0.001 |
| *Bleeding Disorder* | 1.59 | (1.29, 1.95) | <0.001 |
| *Systemic Infection* | 1.57 | (1.02, 2.44) | 0.040 |
|  |  |  |  |

**Table S6 – THA multivariate regression model results for transfusion**

| **Variable** | **OR** | **CI** | **P val** |
| --- | --- | --- | --- |
| Race |  |  |  |
| *Black* | 1.19 | (1.11, 1.27) | <0.001 |
| *Hispanic* | 1.33 | (1.21, 1.46) | <0.001 |
| *Other* | 1.13 | (1.01, 1.27) | 0.033 |
| *Missing* | 0.88 | (0.83, 0.93) | <0.001 |
| Female | 1.96 | (1.88, 2.04) | <0.001 |
| Age (yrs) | 1.01 | (1.01, 1.02) | <0.001 |
| BMI | 0.95 | (0.95, 0.95) | <0.001 |
| ASA | 1.72 | (1.66, 1.78) | <0.001 |
| Operative Time | 1.14 | (1.13, 1.14) | 0 |
| Med History |  |  |  |
| *Smoker* | 0.85 | (0.8, 0.9) | <0.001 |
| *HTN* | 1.15 | (1.1, 1.19) | <0.001 |
| *CHF* | 1.67 | (1.37, 2.04) | <0.001 |
| *DM* | 1.12 | (1.06, 1.18) | <0.001 |
| *Dialysis* | 2.62 | (2.11, 3.26) | <0.001 |
| *Malignancy* | 2.11 | (1.75, 2.54) | <0.001 |
| *Systemic Steroids* | 1.21 | (1.11, 1.31) | <0.001 |
| *Bleeding Disorder* | 2.26 | (2.07, 2.45) | <0.001 |
| *Systemic Infection* | 1.75 | (1.47, 2.08) | <0.001 |

**Table S7 – THA multivariate regression model results for discharge to facility**

| **Variable** | **OR** | **CI** | **P val** |
| --- | --- | --- | --- |
| Race |  |  |  |
| *Black* | 1.73 | (1.66, 1.81) | <0.001 |
| *Hispanic* | 1.6 | (1.49, 1.71) | <0.001 |
| *Other* | 0.8 | (0.73, 0.88) | <0.001 |
| *Missing* | 0.4 | (0.39, 0.42) | 0 |
| Female | 1.83 | (1.78, 1.88) | 0 |
| Age (yrs) | 1.08 | (1.08, 1.08) | 0 |
| BMI | 1.01 | (1.01, 1.01) | <0.001 |
| ASA | 1.77 | (1.72, 1.81) | 0 |
| Operative Time | 1.07 | (1.06, 1.07) | 0 |
| Med History |  |  |  |
| *Smoker* | 1.46 | (1.4, 1.52) | <0.001 |
| *HTN* | 1.08 | (1.05, 1.11) | <0.001 |
| *CHF* | 1.63 | (1.38, 1.92) | <0.001 |
| *DM* | 1.23 | (1.19, 1.28) | <0.001 |
| *Dialysis* | 2.67 | (2.19, 3.27) | <0.001 |
| *Malignancy* | 1.65 | (1.39, 1.96) | <0.001 |
| *Systemic Steroids* | 1.3 | (1.23, 1.38) | <0.001 |
| *Bleeding Disorder* | 1.57 | (1.47, 1.69) | <0.001 |
| *Systemic Infection* | 2.55 | (2.21, 2.95) | <0.001 |

**Table S8 – TKA multivariate regression model results for major complication**

| **Variable** | **OR** | **CI** | **P val** |
| --- | --- | --- | --- |
| Race |  |  |  |
| *Black* | 1.25 | (1.16, 1.34) | <0.001 |
| *Hispanic* | 1.13 | (1.03, 1.24) | 0.008 |
| *Other* | 0.8 | (0.69, 0.92) | 0.002 |
| *Missing* | 1.13 | (1.07, 1.2) | <0.001 |
| Female | 0.82 | (0.79, 0.86) | <0.001 |
| Age (yrs) | 1.02 | (1.02, 1.02) | <0.001 |
| BMI | 1.01 | (1.01, 1.01) | <0.001 |
| ASA | 1.3 | (1.25, 1.35) | <0.001 |
| Operative Time | 1.03 | (1.03, 1.04) | <0.001 |
| Med History | |  |  |
| *Smoker* | 1.32 | (1.22, 1.41) | <0.001 |
| *HTN* | 1.1 | (1.04, 1.15) | <0.001 |
| *CHF* | 1.71 | (1.33, 2.21) | <0.001 |
| *DM* | 1.09 | (1.04, 1.15) | <0.001 |
| *Dialysis* | 2.05 | (1.48, 2.85) | <0.001 |
| *Malignancy* | 1.46 | (0.9, 2.37) | 0.122 |
| *Systemic Steroids* | 1.42 | (1.29, 1.56) | <0.001 |
| *Bleeding Disorder* | 1.63 | (1.46, 1.82) | <0.001 |
| *Systemic Infection* | 2.45 | (1.8, 3.33) | <0.001 |

**Table S9 – TKA multivariate regression model results for mortality**

| **Variable** | **OR** | **CI** | **P val** |
| --- | --- | --- | --- |
| Race |  |  |  |
| *Black* | 1.27 | (0.85, 1.9) | 0.233 |
| *Hispanic* | 1.25 | (0.78, 1.98) | 0.348 |
| *Other* | 0.89 | (0.44, 1.83) | 0.757 |
| *Missing* | 0.84 | (0.59, 1.19) | 0.323 |
| Female | 0.63 | (0.5, 0.79) | <0.001 |
| Age (yrs) | 1.08 | (1.06, 1.09) | <0.001 |
| BMI | 1.02 | (1, 1.04) | 0.036 |
| ASA | 2.14 | (1.72, 2.67) | <0.001 |
| Operative Time | 1.03 | (1, 1.05) | 0.031 |
| Med History | |  |  |
| *Smoker* | 1.7 | (1.15, 2.52) | 0.007 |
| *HTN* | 1.11 | (0.84, 1.47) | 0.462 |
| *CHF* | 1.55 | (0.57, 4.26) | 0.387 |
| *DM* | 1.45 | (1.13, 1.86) | 0.003 |
| *Dialysis* | 6.05 | (2.74, 13.38) | <0.001 |
| *Malignancy* | 5.02 | (1.53, 16.4) | 0.007 |
| *Systemic Steroids* | 1.84 | (1.19, 2.87) | 0.006 |
| *Bleeding Disorder* | 1.71 | (1.09, 2.7) | 0.019 |
| *Systemic Infection* | 5.23 | (1.9, 14.37) | 0.001 |

**Table S10 – TKA multivariate regression model results for return to OR**

| **Variable** | **OR** | **CI** | **P val** |
| --- | --- | --- | --- |
| Race |  |  |  |
| *Black* | 1.13 | (1.01, 1.27) | 0.032 |
| *Hispanic* | 0.99 | (0.85, 1.15) | 0.876 |
| *Other* | 0.65 | (0.51, 0.84) | <0.001 |
| *Missing* | 0.98 | (0.89, 1.08) | 0.690 |
| Female | 0.75 | (0.7, 0.8) | <0.001 |
| Age (yrs) | 1.01 | (1, 1.01) | <0.001 |
| BMI | 1.01 | (1.01, 1.02) | <0.001 |
| ASA | 1.41 | (1.32, 1.5) | <0.001 |
| Operative Time | 1.05 | (1.04, 1.05) | <0.001 |
| Med History | |  |  |
| *Smoker* | 1.68 | (1.52, 1.86) | <0.001 |
| *HTN* | 1.16 | (1.08, 1.26) | <0.001 |
| *CHF* | 1.7 | (1.16, 2.5) | 0.006 |
| *DM* | 1.02 | (0.94, 1.11) | 0.615 |
| *Dialysis* | 2.1 | (1.29, 3.41) | 0.002 |
| *Malignancy* | 1.54 | (0.75, 3.16) | 0.231 |
| *Systemic Steroids* | 1.55 | (1.34, 1.78) | <0.001 |
| *Bleeding Disorder* | 1.62 | (1.37, 1.92) | <0.001 |
| *Systemic Infection* | 2.15 | (1.33, 3.46) | 0.002 |

**Table S11 – TKA multivariate regression model results for VTE**

| **Variable** | **OR** | **CI** | **P val** |
| --- | --- | --- | --- |
| Race |  |  |  |
| *Black* | 1.45 | (1.3, 1.63) | <0.001 |
| *Hispanic* | 1.3 | (1.14, 1.49) | <0.001 |
| *Other* | 0.96 | (0.78, 1.17) | 0.663 |
| *Missing* | 1.2 | (1.09, 1.31) | <0.001 |
| Female | 0.97 | (0.91, 1.04) | 0.405 |
| Age (yrs) | 1.02 | (1.01, 1.02) | <0.001 |
| BMI | 1.01 | (1, 1.01) | 0.03 |
| ASA | 1.02 | (0.95, 1.08) | 0.634 |
| Operative Time | 1.02 | (1.01, 1.03) | <0.001 |
| Med History | |  |  |
| *Smoker* | 0.92 | (0.81, 1.04) | 0.175 |
| *HTN* | 0.93 | (0.87, 1) | 0.050 |
| *CHF* | 1.12 | (0.67, 1.88) | 0.668 |
| *DM* | 0.98 | (0.9, 1.07) | 0.646 |
| *Dialysis* | 1.1 | (0.54, 2.22) | 0.799 |
| *Malignancy* | 1.55 | (0.73, 3.3) | 0.254 |
| *Systemic Steroids* | 1.16 | (0.99, 1.36) | 0.069 |
| *Bleeding Disorder* | 1.41 | (1.17, 1.7) | <0.001 |
| *Systemic Infection* | 1.61 | (0.9, 2.88) | 0.102 |

**Table S12 – TKA multivariate regression model results for surgical site complications**

| **Variable** | **OR** | **CI** | **P val** |
| --- | --- | --- | --- |
| Race |  |  |  |
| *Black* | 0.97 | (0.85, 1.11) | 0.690 |
| *Hispanic* | 1.03 | (0.87, 1.22) | 0.707 |
| *Other* | 0.73 | (0.55, 0.96) | 0.024 |
| *Missing* | 2.18 | (2, 2.38) | <0.001 |
| Female | 0.8 | (0.75, 0.86) | <0.001 |
| Age (yrs) | 1 | (0.99, 1) | 0.163 |
| BMI | 1.03 | (1.03, 1.04) | <0.001 |
| ASA | 1.47 | (1.37, 1.58) | <0.001 |
| Operative Time | 1.04 | (1.04, 1.05) | <0.001 |
| Med History | |  |  |
| *Smoker* | 1.94 | (1.75, 2.15) | <0.001 |
| *HTN* | 1.17 | (1.08, 1.27) | <0.001 |
| *CHF* | 1.87 | (1.24, 2.81) | 0.002 |
| *DM* | 1.01 | (0.92, 1.1) | 0.841 |
| *Dialysis* | 1.82 | (1.01, 3.28) | 0.044 |
| *Malignancy* | 0.45 | (0.11, 1.86) | 0.268 |
| *Systemic Steroids* | 1.56 | (1.34, 1.82) | <0.001 |
| *Bleeding Disorder* | 1.43 | (1.17, 1.74) | <0.001 |
| *Systemic Infection* | 2 | (1.19, 3.38) | 0.009 |

**Table S13 – TKA multivariate regression model results for transfusion**

| **Variable** | **OR** | **CI** | **P val** |
| --- | --- | --- | --- |
| Race |  |  |  |
| *Black* | 1.13 | (1.06, 1.21) | <0.001 |
| *Hispanic* | 0.99 | (0.92, 1.08) | 0.859 |
| *Other* | 0.72 | (0.64, 0.81) | <0.001 |
| *Missing* | 0.81 | (0.76, 0.86) | <0.001 |
| Female | 1.58 | (1.52, 1.65) | <0.001 |
| Age (yrs) | 1.02 | (1.02, 1.02) | <0.001 |
| BMI | 0.96 | (0.96, 0.96) | <0.001 |
| ASA | 1.45 | (1.4, 1.5) | <0.001 |
| Operative Time | 1.08 | (1.08, 1.09) | 0 |
| Med History | |  |  |
| *Smoker* | 1 | (0.93, 1.07) | 0.918 |
| *HTN* | 1.13 | (1.08, 1.18) | <0.001 |
| *CHF* | 1.75 | (1.39, 2.2) | <0.001 |
| *DM* | 1.2 | (1.14, 1.25) | <0.001 |
| *Dialysis* | 2.24 | (1.69, 2.96) | <0.001 |
| *Malignancy* | 2.02 | (1.4, 2.91) | <0.001 |
| *Systemic Steroids* | 1.23 | (1.13, 1.34) | <0.001 |
| *Bleeding Disorder* | 1.66 | (1.51, 1.83) | <0.001 |
| *Systemic Infection* | 1.5 | (1.07, 2.11) | 0.018 |

**Table S14 – TKA multivariate regression model results for discharge to facility**

| **Variable** | **OR** | **CI** | **P val** |
| --- | --- | --- | --- |
| Race |  |  |  |
| *Black* | 1.62 | (1.57, 1.68) | <0.001 |
| *Hispanic* | 1.34 | (1.29, 1.39) | <0.001 |
| *Other* | 0.98 | (0.93, 1.03) | 0.469657 |
| *Missing* | 0.34 | (0.33, 0.35) | 0 |
| Female | 1.81 | (1.78, 1.85) | 0 |
| Age (yrs) | 1.08 | (1.07, 1.08) | 0 |
| BMI | 1.03 | (1.03, 1.03) | 0 |
| ASA | 1.37 | (1.35, 1.4) | <0.001 |
| Operative Time | 1.08 | (1.08, 1.08) | 0 |
| Med History | |  |  |
| *Smoker* | 1.29 | (1.24, 1.33) | <0.001 |
| *HTN* | 1.05 | (1.02, 1.07) | <0.001 |
| *CHF* | 1.69 | (1.47, 1.94) | <0.001 |
| *DM* | 1.24 | (1.22, 1.27) | <0.001 |
| *Dialysis* | 2.05 | (1.69, 2.48) | <0.001 |
| *Malignancy* | 1.52 | (1.19, 1.94) | <0.001 |
| *Systemic Steroids* | 1.24 | (1.18, 1.3) | <0.001 |
| *Bleeding Disorder* | 1.43 | (1.35, 1.51) | <0.001 |
| *Systemic Infection* | 1.16 | (0.95, 1.42) | 0.146012 |

**Article Title:** Racial disparities in post-operative complications and discharge destination following total joints arthroplasty

**Journal Name:** Archives of Orthopaedic and Trauma Surgery

**Author Names:** Alex Upfill-Brown, MD, MSc; Noah Paisner, BS; Adam Sassoon, MD, MS

**Corresponding Author:** Adam Sassoon, MD, MS, UCLA Department of Orthopaedic Surgery, asassoon@mednet.ucla.edu
